# Data Visualization: A Guide to Visual Storytelling for Libraries

**DOI:** 10.5195/jmla.2018.346

**Published:** 2018-01-02

**Authors:** Nathalie Reid

A compilation of twelve chapters, *Data Visualization: A Guide for Visual Storytelling for Libraries* provides an introduction for librarians on how they can effectively communicate through visualizing how their collections and services are being used. The book is divided into three sections: (1) choosing and interpreting datasets for visualization, (2) tools and technologies for creating meaningful visualizations, and (3) case studies of information visualization projects or applications in libraries.

Through case studies, the book illustrates that a number of technologies can be used to create these visualization such as Google Charts, Excel, and R programming language. The unique method of framing the book through case studies demonstrates how storytelling through data does not have to be dry and boring simply through statistics but can instead be visually captivating and engaging to its audience.

The initial chapters in the book provide background in data visualization. For example, in chapter one, Phetteplace introduces readers to critical skills needed for doing data visualization such as acquiring, parsing, filtering, mining, representing, refining, and interacting with the data. In chapter two, Zoss goes through different types of charts and discusses the strengths and weakness of each, offering readers information that is needed for deciding which chart will work best for their datasets. Other chapters, such as chapter four, “Using Google Tools to Create Public Analytics Visualizations,” provide hands-on guidance for a specific tool in creating designs.

The book includes twenty pages of colored visualizations such as pie charts, bar charts, line charts, scatterplots, choropleth maps, waffle charts, word clouds, infographics, organization charts, radial charts, and tree charts. Many of the charts list which software was used to create the chart.

This work is incredibly useful for librarians who want to showcase their roles in a number of areas including research activity, teaching of information literacy, and collection development. It would be of most use to public, academic, and special librarians in their work to demonstrate the services that they provide to users.

While other comparable data visualization books exist, none is specific to libraries. As libraries become more dependent on data to monitor usage and justify services, this book fills a gap in the literature by addressing data visualization in a library context. It is of special interest in that it also includes case examples of how data visualization was used in specific libraries for an institutional repository and in another case for an archival collection. This volume also includes an appendix that lists and describes all visualization technologies discussed in the book for easy reference. At the end of the book, there are brief descriptions of all the contributing authors.

This book is highly recommended for libraries that collect data and want to make their statistics more visually compelling and add a story layer to their data.

